# A Short Performance Anxiety Scale for Musicians

**DOI:** 10.3389/fpsyg.2021.781262

**Published:** 2022-01-26

**Authors:** Isabella Mazzarolo, Emery Schubert

**Affiliations:** Empirical Musicology Laboratory, School of the Arts & Media, University of New South Wales, Sydney, NSW, Australia

**Keywords:** performance anxiety, music, psychometrics, experience, severity, avoidance, frequency, intensity

## Abstract

Given the prevalence of debilitating anxiety associated with music performance, there is a need for rapid, pinpointed assessment of the extent to which an individual experiences music performance anxiety (MPA). A short, five item scale, the Mazzarolo Music Performance Anxiety Scale (M-MPAS), was developed to capture retrospective self-reported estimates of the frequency, intensity and aversion tendency associated with performing music. 102 musicians completed the scale, as well as an established MPA inventory. The M-MPAS was found to be internally reliable (Cronbach’s alpha = 0.894) with all items being statistically relevant to the overall scale. Furthermore, the M-MPAS was correlated with the relevant factors of an established MPA measure (*r* = 0.791), suggesting that the new scale exhibited good construct validity. M-MPAS is easy to score, with a scale range of 0–30. A score of 11 or above is suggested as the range in which a diagnosis of potential high MPA can be made, but more research into this and the psychometric robustness of the scale is called for. Nevertheless, the reliability and validity demonstrated in the present study, along with the brevity of the M-MPAS suggest that the newly proposed scale may offer considerable triaging benefits for pinpointed assessment of the extent to which an individual experiences MPA.

## Introduction

Music performance anxiety (MPA) is “the experience of persisting, distressful apprehension and/or actual impairment of performance skills in a public context, to a degree unwarranted given the individual’s musical aptitude, training, and level of preparation” (Salmon, 1990, p. 3). The condition affects 15–25% of musicians, with even higher rates in younger musicians (Robson and Kenny, 2017; Fernholz et al., 2019). This makes the diagnosis and management of MPA a key concern that has attracted considerable research interest. There are currently several psychometric tools that assess MPA, but there has been a neglect in the rapid assessment of the nature of MPA episodes, an issue this paper proposes to address through the reporting of a newly developed instrument.

In his review of MPA literature, Taborsky (2007) proposed that “numerous factors may influence the intensity of anxiety, often resulting in increased heart rate, state anxiety, and other physical symptoms” (p. 22). But Taborsky’s review reveals that the perception of the *global intensity* of MPA is not a significant matter in the design of the MPA measures currently available. While psychometric methods require a broad sweep of various, specific response items to facilitate a good estimate of the psychological construct under investigation, it is still surprising that direct questioning of global MPA intensity is not requested. There is compelling literature on small and even single-item measures as providing good approximations of aspects of psychometric constructs, particularly as initial screening tools (Williams and Smith, 2018; Casu and Gremigni, 2019; Turon et al., 2019). Otherwise, the psychometric scale relies on gathering data regarding a large number of possible individual facets that contribute to the MPA experience, and in doing so could become inordinately long, potentially redundant, or risk missing one or more key facets even before a diagnosis of MPA is made. As shown in Table 1, the most frequently used MPA assessment tools require between 15 and 58 items to be rated by the individual. A direct, simple interrogation of global MPA intensity would by-pass the need for such specificity as an initial step, and return to the root of MPA, which, as Salmon’s (1990) definition infers, is concerned with *experience*.

**TABLE 1:** Sample of commonly cited, published MPA measures.

Source	Name of measure	Items	Components	Relevant global measures*	Notes
Brodsky et al. (1994)	Music Performance Stress Survey (MPSS)	25	(1) Physical conditions, (2) Related symptoms, (3) Involvement in interventions for musicians’ occupational medical and psychological problems, (4) Somatic symptoms, (5) Emotional attitudes (6) Effects on career	a, f, i	Psychometric properties not reported
Cırakoğlu and Şentürk (2013)	Performance Anxiety Scale for Music Students (PASMS)	24	(1) Fear of stage, (2) Avoidance, (3) Symptoms	a	
Kenny et al. (2004)	Kenny Music Performance Anxiety Inventory (K-MPAI – also the K-MPAI-R)	40	(1) Proximal somatic anxiety and worry about performance, (2) Worry/dread (Negative cognitions) focused on self/other scrutiny, (3) Depression/hopelessness (Psychological vulnerability), (4) Parental empathy, (5) Memory, (6) Generational transmission of anxiety, (7) Anxious apprehension, (8) Biological vulnerability	a	
Lehrer et al. (1990)	Music Performance Anxiety Questionnaire (MPAQ)	32	(1) Planning to cope with anxiety symptoms, (2) High standards and a judgmental attitude about performance, worry about anxiety and its (3) Effects on performance, (4) Concern with the reactions of important others, (5) Concern about distraction in oneself and in the audience	a	
Osborne and Kenny (2005)	Music Performance Anxiety Inventory for Adolescents (MPAI-A)	15	(1) Somatic, (2) Cognitive, (3) Behavioral	a	Anxiety experienced by adolescent musicians
Papageorgi (2007, 2021)	Adolescent Musicians’ Performance Anxiety Scale (AMPAS)	20	(1) Negative outcome expectancies, (2) Negative experiences in performance, (3) Evidence of pre-evaluation anxiety, (4) Experience of physiological symptoms of anxiety, (5) Concern about others’ judgments, (6) Negative perception of anxiety	a, f	
Sheriff and Yoong (2015)	Music Performance Anxiety Scale (MPAS)	58	(1) Causes/Situational factors; (2) Temporal occurrence; (3) Cognitive manifestations; (4) Affective manifestations; (5) Behavioral manifestations; (6) Somatic manifestations; (7) Autonomic Arousal	a, f, i	More validation required; scale is not publicly available
Wolfe (1989)	Music Performance Anxiety Scale (MPAS)	55	(1) Adaptive anxiety (helpful); (2) Maladaptive anxiety (harmful); (3) Music performance anxiety: (3a) Cognitive and (3b) Emotional components)	a	

*None of the measures contained items explicitly about global frequency, intensity, and few contained items explicitly on global aversion (of music performance).

*Where some measure of these qualities were identified, they are labeled accordingly: f, frequency; i, intensity; a, music performance aversion. For more detailed overviews of MPA measures, see, for example, Osborne and Kenny (2005), Goren (2014), Burin and Osório (2016), Brugués (2018).*

Of course, intensity alone is not sufficient to capture the experience of MPA. If an MPA episode is particularly long, then this would increase the MPA experience in comparison to a short MPA episode. Similarly, if MPA episodes occur frequently, at a given level of intensity, the overall experience of MPA would be more problematic than, for example, a single episode among many performances. A small number of MPA measures (Papageorgi, 2007, AMPAS; Papageorgi, 2021, MPAS; Sheriff and Yoong, 2015) have been cited that gather data about frequency of aspects of MPA episodes, however none have been cited that explicitly probe global indication of MPA experience (see Table 1). Frequency and intensity information is commonly gathered in extant (non-MPA) psychometric scales, both as simple global scales, and as fully fledged psychometric dimensions (e.g., Swain and Jones, 1993; Wisniewski et al., 2006; Kozel et al., 2008; Walker et al., 2010; Schneider and Stone, 2014; Van Lancker et al., 2016).

The studies cited that gather responses on global items tend also to include a third concept, which can be characterized as the burden of the experience, in other words, the extent to which the experience (in this case of MPA) creates a sense of seeking to avoid future such experiences. After all, one individual may experience considerable negative impact of MPA even with relatively low frequency and intensity of episodes while another may experience less negative impact even with the same frequency and intensity of episodes. Measuring the aversiveness tendency of the MPA experience was therefore considered essential. An early example of an MPA measure that explicitly makes this distinction is the MPAS (Wolfe, 1989; see Table 1), where helpful, adaptive anxiety and unhelpful, maladaptive anxiety were included as separate factors. This can be summarized in global terms as the impact that MPA has on aversion from performing, a commonly reported factor in MPA measures.

While such simple, global items may allow for rapid assessment of MPA, we acknowledge that they cannot replace comprehensive, multidimensional diagnostic tools. But the availability of global indications of MPA, and consequent rapid assessment of MPA experience would serve the research and clinical community. Furthermore, very short assessment tools can be sufficiently reliable, making their value potentially high. This paper therefore takes a first step in developing a short scale that assesses MPA experience. The aim of the study was to present a first step toward the rapid, reliable and pinpointed initial measure of MPA experience.

## Methods

### Participants

A total of 102 musicians (37 male and 65 female) from Australia volunteered to participate in this study by completing one online questionnaire. 79 were current students, studying music in a conservatoire or tertiary setting. The remaining 23 participants were former music students who were not currently studying music at a tertiary level. The sample was composed of 33 pianists, 22 woodwind players, 15 vocalists, 13 guitarists/bass guitarists, 8 string players, 6 percussionists, 4 brass players, and 1 harpist. Participants’ ages ranged from 18 to 60 years, with an average of 24.39 years (*SD* = 8.49). Participants had learned their instrument for an average of 11.23 years (*SD* = 4.76).

### Materials

#### Mazzarolo Music Performance Anxiety Scale

Self-reported MPA was measured using a newly developed five-item scale – the Mazzarolo Music Performance Anxiety Scale (M-MPAS). By covering the areas of intensity, frequency, and aversion, these questions aimed to enable a simple, quick, and effective way to measure MPA. The development of the scale followed the line of argument presented in the Introduction, with additional items added to allow reverse scoring and to emphasize the critical outcome of MPA, aversion of performance. The scale uses a seven-point Likert-type scale ranging from 0 = “Strongly disagree” to 6 = “Strongly agree” in response to five statements: (1) for each of the five items: “I experience strong nerves/anxiety before I perform,” (intensity) (2) “I frequently experience nerves/anxiety before I perform (frequency),” (3) “I avoid performing in order to alleviate my nerves/anxiety,” (aversion), (4) “I feel positive before my music performances,” (“negative” aversion, with reverse scoring used for this statement), and (5) “I don’t want to go ahead with my music performances because of my nerves/anxiety” (aversion) (see Table 2).

**TABLE 2:** Mazzarolo Music Performance Anxiety Scale (M-MPAS).

	Strongly disagree	Disagree	Slightly disagree	Neither agree nor disagree	Slightly agree	Agree	Strongly agree
1. I experience strong nerves/anxiety before I perform	0	1	2	3	4	5	6
2. I frequently experience nerves/anxiety before I perform	0	1	2	3	4	5	6
3. I avoid performing in order to alleviate my nerves/anxiety	0	1	2	3	4	5	6
4. I feel positive before my music performances	6	5	4	3	2	1	0
5. I don’t want to go ahead with my music performances because of my nerves/anxiety	0	1	2	3	4	5	6

*After an introduction, the M-PAS items list is preceded by the following instruction: “The following statements will ask you about your experience with music performance anxiety. To what extent do you disagree or agree with each of the following statements?”.*

*Numerals in the matrix are not shown to the participant/client, but are used for scoring by the researcher/therapist. The score is calculated as a simple sum of the 5 numerals. Scores can range from 0 (lowest MPA experience) to 30 (highest MPA experience).*

#### Kenny Music Performance Anxiety Inventory

The K-MPAI was used to assess the construct validity of the M-MPAS. It was selected because it is in wide usage among researchers, is considered reasonably comprehensive and has strong psychometric properties (Chang-Arana et al., 2018; see also Table 1). It consists of 40 items and encompasses eight dimensions of MPA: (1) Proximal somatic anxiety and worry about performance, (2) Worry/dread (Negative cognitions) focused on self/other scrutiny, (3) Depression/hopelessness (Psychological vulnerability), (4) Parental empathy, (5) Memory, (6) Generational transmission of anxiety, (7) Anxious apprehension, and (8) Biological vulnerability. Of these, factors 1, 2, 3, and 7 are most directly related to the *experience* of MPA, which we argue are necessary for diagnosis, while 4, 5, 6, and 8 are concerned with etiology. The items of the K-MPAI are answered on a seven-point Likert-type scale (0 = “Strongly disagree” to 6 = “Strongly agree”). Answers with a higher score indicate greater anxiety and psychological distress in the context of music performance.

### Procedure

The Human Research Ethics Advisory Panel of UNSW Australia approved the study. Students studying Music were invited to participate in the study via email. These students were encouraged to forward the email to any other musicians over the age of 18 who might be interested in participating. Participants entered data using Qualtrics survey software^1^. They were informed that their answers would be de-identified and only be used for research purposes. To reduce bias due to order effects, factor presentation order of the K-MPAI was randomized. In addition to completing the M-MPAS and K-MPAI, participants provided background information and completed other tasks that are not reported here.

## Results

### Internal Consistency of the Mazzarolo Music Performance Anxiety Scale

Cronbach’s alpha was calculated with the M-MPAS items to examine the reliability of the instrument. The results revealed a Cronbach’s alpha value of 0.894, which indicates good internal consistency within the M-MPAS (Ponterotto and Ruckdeschel, 2007). The Item-total Statistics table presents the value that Cronbach’s alpha would be if a particular question was deleted from the scale. Table 3 shows that the removal of any question would result in a lower Cronbach’s alpha. Since all such models produce lower Cronbach’s alpha values, none of the questions within the M-MPAS should be removed, though Question 4 is the worst performing (Cronbach’s alpha of the scale = 0.888 when omitted).

**TABLE 3:** M-MPAS item-total statistics.

M-MPAS statements	Scale mean if item deleted	Scale variance if item deleted	Corrected item-total correlation	Squared multiple correlation	Cronbach’s alpha if item deleted
1. I experience strong nerves/anxiety before I perform	11.39	41.053	0.808	0.700	0.857
2. I frequently experience nerves/anxiety before I perform	11.05	43.73	0.693	0.596	0.881
3. I avoid performing in order to alleviate my nerves/anxiety	12.89	37.206	0.79	0.665	0.862
4. I feel positive before my music performances	12.59	46.165	0.663	0.443	0.888
5. I don’t want to go ahead with my music performances because of my nerves/anxiety	12.98	39.128	0.773	0.663	0.864

*Cronbach’s alpha = 0.894.*

### Construct Validity of Mazzarolo Music Performance Anxiety Scale

A Pearson product-moment correlation was performed between the M-MPAS and the scores of items taken from 4 factors of the K-MPAI that investigated MPA symptoms (proximal, worry/dread, memory, and anxious apprehension). Two responses could not be used for this analysis as those belonged to participants who had not completed the K-MPAI. The results showed that there was a strong, positive correlation between the reduced K-MPAI and M-MPAS scores (*r* = 0.797, *n* = 100, *p* < 0.001) and had a large effect size (Cohen, 1992).

### Diagnostic Criteria

Cut-off publications for K-MPAI vary. Paliaukiene et al. (2018) used scores above 130 to designate a “high MPA” group. This figure was based on the principle of one standard deviation above the mean. For clinical diagnosis, based on Youden’s Index, 104 has also been proposed (Kenny, 2015). Therefore the cut-off above which high MPA would be initially diagnosed via the M-MPAS was estimated through a linear regression with the overall K-MPAI score for the four experience related factors. This score was scaled from a range of 0–148 (using 24 items) to a range of 0–240 (for the 40 items upon which published cut-offs are based). The least squares linear regression equation to estimate the conversion from K-MPAI to the M-MPAS for the present dataset explained 62.89% of the variance, and can be expressed as:


M-M⁢P⁢A⁢S=0.1444×K-MPAI-4.0623

The K-MPAI cut-off of 104 (out of 240) can therefore be estimated as 11.0 (out of 30) on the M-MPAS.

## Discussion and Conclusion

The M-MPAS was developed to address the need for a rapid, preliminary assessment of an individual’s Music Performance Anxiety (MPA). The five item scale gathers data on the global frequency and intensity of MPA episodes, as well as on the negative impact (aversion to future music performance) of the experience, and deliberately avoids matters concerned with etiology. The correlation analyses revealed that a strong relationship existed between the relevant, experience focused factors of the K-MPAI, suggesting strong construct validity of the M-MPAS (Cook, 1979). These K-MPAI factors may be viewed as capturing in greater depth the essence of the MPA experience, but consist of more items and time requirements than the M-MPAS (24 versus 5 items). Furthermore, we made an initial estimate of the cut-off above which an individual may be diagnosed as having high MPA. The five item scale has a possible range of 0–30, with scores at or above 11 proposed as an indicator of potential high MPA. The study has several limitations, and will need further investigation before it could be adopted for clinical use. For example, the question wording for three of the items (1, 3 and 4) referred to feelings experienced prior to, rather than during, performance. Ideally information about different stages of the performance episodes would be desirable, but for the sake of clarity, brevity and simplicity, we chose the antecedent time framing as the point of focus for these items. The reliability and validity analyses suggest this limitation is tolerable, but future work may seek to investigate the psychometric impact of modifications that identify and improve possible weaknesses of the scale. Access to an instrument that allows rapid, initial assessment of MPA is a matter of urgency given the prevalence of performance anxiety in a discipline that should above all be bringing joy and passion to its creators.

## Data Availability Statement

The datasets presented in this article are not readily available because the ethics committee has not approved distribution of the data collected. Requests to access these datasets should be directed to Emery Schubert, e.schubert@unsw.edu.au.

## Ethics Statement

The studies involving human participants were reviewed and approved by the Human Research Ethics Advisory Panel of UNSW Australia. The patients/participants provided their written informed consent to participate in this study.

## Author Contributions

IM designed the study, collected and analyzed the data, and wrote the drafts of the manuscript. ES assisted with the design of the study, analysis, and editing of the manuscript. Both authors were involved in determining the concept and rationale of the study. The study commenced while IM was conducting Honors research and considerably reworked as part of her Doctoral research program.

## Conflict of Interest

The authors declare that the research was conducted in the absence of any commercial or financial relationships that could be construed as a potential conflict of interest.

## Publisher’s Note

All claims expressed in this article are solely those of the authors and do not necessarily represent those of their affiliated organizations, or those of the publisher, the editors and the reviewers. Any product that may be evaluated in this article, or claim that may be made by its manufacturer, is not guaranteed or endorsed by the publisher.

## Footnotes

1
www.qualtrics.com

## References

[B1] Brodsky W. Sloboda J. A. Waterman M. G. (1994). An exploratory investigation into auditory style as a correlate and predictor of music performance anxiety. *Med. Probl. Perform. Art.* 9 101–112.

[B2] Brugués A. O. (2018). *Music Performance Anxiety: A Comprehensive Update of the Literature.* Newcastle-upon-Tyne: Cambridge Scholars Publisher.

[B3] Burin A. B. Osório F. D. L. (2016). Interventions for music performance anxiety: results from a systematic literature review. *Rev. Psiquiatr. Clín.* 43 116–131. 10.1590/0101-60830000000097

[B4] Casu G. Gremigni P. (2019). Is a single-item measure of self-rated mental health useful from a clinimetric perspective? *Psychother. Psychosom.* 88 177–178. 10.1159/000497373 30909284

[B5] Chang-Arana ÁM. Kenny D. T. Burga-León A. A. (2018). Validation of the Kenny Music Performance Anxiety Inventory (K-MPAI): a cross-cultural confirmation of its factorial structure. *Psychol. Music* 46 551–567. 10.1177/0305735617717618

[B6] Cırakoğlu O. C. Şentürk G. C. (2013). Development of a performance anxiety scale for music students. *Med. Probl. Perform. Art.* 28 199–206. 24337031

[B7] Cohen J. (1992). A power primer. *Psychol. Bull.* 112 155–159. 19565683 10.1037//0033-2909.112.1.155

[B8] Cook T. D. (1979). *Quasi-Experimentation: Design & Analysis Issues for Field Settings.* Boston, MA: Houghton Mifflin.

[B9] Fernholz I. Mumm J. L. M. Plag J. Noeres K. Rotter G. Willich S. N. (2019). Performance anxiety in professional musicians: a systematic review on prevalence, risk factors and clinical treatment effects. *Psychol. Med.* 49 2287–2306. 10.1017/S0033291719001910 31474244

[B10] Goren L. (2014). *A Meta-Analysis of Nonpharmacologic Psychotherapies for Music Performance Anxiety* (Psy.D.). Doctoral dissertation. San Francisco, CA: California Institute of Integral Studies.

[B11] Kenny D. T. (2015). *Identifying Cut-Off Scores for Clinical Purposes for the Kenny Music Performance Anxiety Inventory (K-MPAI) in a Population of Professional Orchestral Musicians. Technical Report.* Available online at: https://www.researchgate.net/publication/282735405_Identifying_cut-off_scores_for_clinical_purposes_for_the_Kenny_Music_Performance_Anxiety_Inventory_K-MPAI_in_a_population_of_professional_orchestral_musicians

[B12] Kenny D. T. Davis P. Oates J. (2004). Music performance anxiety and occupational stress amongst opera chorus artists and their relationship with state and trait anxiety and perfectionism. *J. Anxiety Disord.* 18:757. 10.1016/j.janxdis.2003.09.004 15474851

[B13] Kozel F. A. Trivedi M. H. Wisniewski S. R. Miyahara S. Husain M. M. Fava M. (2008). Treatment outcomes for older depressed patients with earlier versus late onset of first depressive episode: a Sequenced Treatment Alternatives to Relieve Depression (STAR*D) report. *Am. J. Geriatr. Psychiatry* 16 58–64. 10.1097/JGP.0b013e31815a43d7 18165462

[B14] Lehrer P. M. Goldman N. S. Strommen E. F. (1990). A principal components assessment of performance anxiety among musicians. *Med. Probl. Perform. Art.* 5 12–18.

[B15] Osborne M. S. Kenny D. T. (2005). Development and validation of a music performance anxiety inventory for gifted adolescent musicians. *J. Anxiety Disord.* 19 725–751. 10.1016/j.janxdis.2004.09.002 16076421

[B16] Paliaukiene V. Kazlauskas E. Eimontas J. Skeryte-Kazlauskiene M. (2018). Music performance anxiety among students of the academy in Lithuania. *Music Educ. Res.* 20 390–397. 10.1080/14613808.2018.1445208

[B17] Papageorgi I. (2007). *Understanding Performance Anxiety in the Adolescent Musician.* Doctoral thesis. London: UCL Institute of Education.

[B18] Papageorgi I. (2021). Typologies of adolescent musicians and experiences of performance anxiety among instrumental learners. *Front. Psychol.* 12:1607. 10.3389/fpsyg.2021.645993 34025516 PMC8136414

[B19] Ponterotto J. G. Ruckdeschel D. E. (2007). An overview of coefficient alpha and a reliability matrix for estimating adequacy of internal consistency coefficients with psychological research measures. *Percept. Mot. Skills* 105 997–1014. 10.2466/pms.105.3.997-1014 18229554

[B20] Robson K. E. Kenny D. T. (2017). Music performance anxiety in ensemble rehearsals and concerts: a comparison of music and non-music major undergraduate musicians. *Psychol. Music.* 45 868–885. 10.1177/0305735617693472

[B21] Salmon P. G. (1990). A psychological perspective on musical performance anxiety: a review of the literature. *Med. Probl. Perform. Art.* 5 2–11.

[B22] Schneider S. Stone A. A. (2014). Distinguishing between frequency and intensity of health-related symptoms from diary assessments. *J. Psychosomatic Res.* 77 205–212. 10.1016/j.jpsychores.2014.07.006 25149030 PMC4265366

[B23] Sheriff F. H. M. Yoong C. Y. (2015). Development of the music performance anxiety scale. *Int. J. Acad. Res. Reflec.* 3 1–9.

[B24] Swain A. Jones G. (1993). Intensity and frequency dimensions of competitive state anxiety. *J. Sports Sci.* 11 533–542. 10.1080/02640419308730024 8114179

[B25] Taborsky C. (2007). Musical performance anxiety: a review of literature. *Update Appl. Res. Music Educ.* 26 15–25. 10.1177/87551233070260010103

[B26] Turon H. Carey M. Boyes A. Hobden B. Dilworth S. Sanson-Fisher R. (2019). Agreement between a single-item measure of anxiety and depression and the hospital anxiety and depression scale: a cross-sectional study. *PLoS One* 14:e0210111. 10.1371/journal.pone.0210111 30608969 PMC6319715

[B27] Van Lancker A. Beeckman D. Verhaeghe S. Van Den Noortgate N. Grypdonck M. Van Hecke A. (2016). An instrument to collect data on frequency and intensity of symptoms in older palliative cancer patients: a development and validation study. *Eur. J. Oncol. Nurs.* 21 38–47. 10.1016/j.ejon.2015.11.003 26952677

[B28] Walker A. J. Gedaly-Duff V. Miaskowski C. Nail L. (2010). Differences in symptom occurrence, frequency, intensity, and distress in adolescents prior to and one week after the administration of chemotherapy. *J. Pediatr. Hematol. Oncol. Nurs.* 27 259–265. 10.1177/1043454210365150 20639347

[B29] Williams G. Smith A. P. (2018). Diagnostic validity of the anxiety and depression questions from the well-being process questionnaire. *J. Clin. Transl. Res.* 4 101–104. 30873498 PMC6412610

[B30] Wisniewski S. R. Rush A. J. Balasubramani G. K. Trivedi M. H. Nierenberg A. A. Stard Investigators (2006). Self-rated global measure of the frequency, intensity, and burden of side effects. *J. Psychiatr. Pract.* 12 71–79. 16728903 10.1097/00131746-200603000-00002

[B31] Wolfe M. L. (1989). Correlates of adaptive and maladaptive musical performance anxiety. *Med. Probl. Perform. Art.* 4 49–56.

